# Enhancing Atrial Fibrillation Risk Prediction in Tobacco-Exposed Individuals: The Role of Pulmonary Function Tests, Symptom Scores, and Imaging

**DOI:** 10.21203/rs.3.rs-6439820/v1

**Published:** 2025-05-07

**Authors:** Nicole Curtis, S. Mehdi Nouraie, Jiantao Pu, Joseph K Leader, Frank C Sciurba, Jessica Bon

## Abstract

**Introduction:** COPD is associated with an increased AFib-related morbidity and mortality. There are several AFib risk prediction models available, but none have been validated in the COPD population. Our study aims to (1) identify spirometric and radiographic variables that are associated with an increased risk of AFib and (2) determine if these associated variables improve the risk discrimination of established AFib risk prediction models in individuals with COPD. **Methods:** We evaluated 755 participants from a single center tobacco-exposed cohort at baseline, 2-, 6-, and 10-year follow up. At each study visit, the following were performed: demographic, medical history, and symptom questionnaires, PFT, and CT imaging. We performed logistic regression analysis to determine cardiopulmonary variables associated with prevalent and incident AFib. The multivariable analysis was adjusted for sex, age, number of pack years, BMI, self-reported heart failure, and anti-hypertensive medication use. Exposure variables that were statistically significant in the logistic regression analysis were added in succession to current AFib risk prediction models, CHA _2_ DS _2_ -VASc and CHARGE-AF, to create updated models. C-statistics were calculated for both risk scores alone as well as with each updated model. **Results:** DLco (OR 0.37, CI 0.16-0.83), heart volume (OR 13.12, CI 2.32-74.17), percentage of emphysema (OR 2.77, CI 1.04-7.40), and mMRC (OR 1.17, CI 1.02-1.35) were associated with prevalent AFib in the multivariable logistic regression analysis. When conducting the discrimination analysis of the AFib risk prediction scores, the addition of these cardiopulmonary variables to CHA _2_ DS _2_ -VASc in incident AFib was found to provide the most profound improvement, from C-statistic 0.51 to 0.70 (p<0.001). **Conclusions:** We identified cardiopulmonary factors associated with an increased risk of AFib in a tobacco-exposed cohort. The incorporation of lung function, CT parameters, and symptom scores in validated AFib prediction models may improve AFib risk discrimination in our chronic lung disease populations. **Trial Registration:** This study was supported by the National Institute of Health (NIH) National Heart, Lung and Blood Institute (NHLBI) grants 1R01HL128289 (J.B.) and P50HL084948 (F.C.S.).

## Introduction

Chronic obstructive pulmonary disease (COPD) is associated with extrapulmonary cardiovascular comorbidities including coronary heart disease, heart failure, and atrial fibrillation (AFib)[1,2]. While the prevalence of AFib in the general adult population is approximately 3%, patients with COPD have a 28% increased risk of developing AFib[3]. Hypoxia, hypercapnia, pulmonary hypertension, electrolyte disturbances, oxidative stress, and chronic systemic inflammation are all proposed processes that contribute to this increased risk[4,5]. In addition to the increased risk of developing AFib, patients with COPD have worse AFib-related morbidity and mortality[4,5,6]. Numerous studies have attempted to understand why COPD is associated with an increased risk of AFib, but there is a paucity of studies evaluating specific lung-related variables via spirometry and imaging.

There are numerous validated and commonly utilized risk prediction models to predict incident AFib, including $CHA_2DS_2$-VASc and the Cohorts for Heart and Aging Research in Genomic Epidemiology AF (CHARGE-AF). Both have not been validated in COPD populations[7,8,9]. A need exists to validate these prediction models in a COPD population, and to determine if factors related to lung disease severity can improve risk discrimination of these models in patients with COPD. Our study aims to (1) identify spirometric and radiographic variables that are associated with an increased risk of AFib and (2) determine if these associated variables improve the risk discrimination of established AFib risk prediction models in individuals with COPD. We hypothesize that spirometric and radiographic variables indicative of worse airflow obstruction, a greater percentage of emphysema, and larger heart volume are associated with increased risk of AFib and will improve risk discrimination of $CHA_2DS_2$-VASc and CHARGE-AF.

### Methods

## Study population

The COPD Specialized Center for Clinically Oriented Research (SCCOR) cohort at the University of Pittsburgh is a prospective observational cohort of 765 current or former smokers with at least a 10 pack-year history of tobacco use. The cohort enrolled participants between July 2007 and December 2012. Exclusion criteria included history of lung cancer, obesity (defined as body mass index (BMI) > 34), and any other significant pulmonary diagnosis. Participants were evaluated at baseline (N = 765), two (N = 393), six (N = 298), and ten year (N = 101) follow up. At each study visit, the following were performed: demographic, medical history, and symptom questionnaires, pulmonary function testing (PFT), and chest computed tomography (CT) imaging. For the current analysis, 10 participants were excluded due to missing data of the primary outcome (self-reported AFib) on medical history questionnaire.

### Study Procedures and Definitions

Prevalent AFib was defined by self-report at the baseline visit. Incident AFib was defined by self-report during any of the follow-up study visits in participants not reporting AFib at baseline. Modified Medical Research Council (mMRC) dyspnea scale was used to assess shortness of breath. mMRC is a tool to assess the severity of breathlessness with scores ranging from 0 (no breathlessness) to 4 (severe breathlessness)[10]. PFTs were performed at each study visit and included pre- and post- bronchodilator spirometry, lung volumes, and diffusion capacity of the lungs for carbon monoxide (DLco). Results were expressed as a percent of the predicted value for healthy individuals of similar age, sex, height, and ethnicity. We utilized the Hankinson, Crapo, and Miller equations for spirometry, lung volumes, and DLco, respectively[11,12,13]. Chest CT scans were obtained at each study visit. The degree of emphysema was determined by measuring the percentage of lung tissue voxels with Hounsfield units (HU) attenuation below – 910 HU. The volume of cardiac and aortic calcifications was calculated using the Agatson score[14]. The diameter of major pulmonary vasculature and heart volume was computed automatically using an artificial intelligence (AI) algorithm[15,16].

$CHA_2DS_2$-VASc and CHARGE-AF were calculated for each participant. $CHA_2DS_2$-VASc was calculated by totaling points for the following: heart failure (1 point), hypertension (1 point), age > 75 (2 points), diabetes (1 point), stroke/transient ischemic attack/thromboembolism (2 points), vascular disease (1 point), age 65–74 (1 point), and female sex (1 point)[8]. Comorbidities were assessed with self-reported medical history questionnaires. CHARGE-AF was calculated utilizing an intricate formula cited in the 2023 ACC/AHA/ACCP/HRS guidelines including the following variables: age, race, height, weight, systolic blood pressure, diastolic blood pressure, smoking status, history of diabetes, and history of myocardial infarction[17].

## Statistical Analysis

Prevalent AFib at baseline and incident AFib during longitudinal follow-up were the primary outcomes of interest. Independent PFT variables included forced expiratory volume in one second ($FEV_1$), forced vital capacity (FVC), $FEV_1$/FVC, residual volume (RV), total lung capacity (TLC), and DLco. Independent radiographic variables included quantitative emphysema, the ratio of pulmonary artery to aorta diameter, heart volume, and volume of vascular calcifications. mMRC was used to quantify symptom severity.

Comparison of baseline characteristics between participants with and without prevalent and incident AFib were evaluated using the independent Student’s t-test for continuous variables and chi-square analysis for categorical variables. Univariate and multivariable logistic regression was performed to determine cardiopulmonary variables associated with prevalent and incident AFib. The multivariable analysis was adjusted for the following: sex, age, number of pack years, BMI, self-reported heart failure, and anti-hypertensive medication use.

Discrimination analysis was performed for both $CHA_2DS_2$-VASc and CHARGE-AF using the area under the time-dependent receiver operating characteristic curve (AUROC). Exposure variables that were statistically significant in the logistic regression analysis were added in succession to these risk prediction scores to create updated models. C-statistic was determined for $CHA_2DS_2$-VASc and CHARGE-AF alone as well as with each updated model.

All statistical analyses were performed via Stata version 18.0[18].

## Results

We evaluated 755 participants from the SCCOR cohort, of which 135 (17.9%) participants reported a history of AFib at the baseline visit (prevalent AFib). During follow-up, an additional 39 (6.3%)

participants reported the development of AFib (incident AFib). A total of 174 participants (23%) had AFib at baseline or during follow-up.

Baseline age, sex, race, and pack years were similar among participants with and without prevalent AFib (Table 1). Those with prevalent AFib had a lower DLco, greater amount of emphysema, worse mMRC score, and larger heart volume. There was a higher percentage of self-reported diabetes, angina, heart failure, thromboembolic disease, hyperlipidemia, and anti-hypertensive medication use in the prevalent AFib group compared to no AFib.

**Table 1. Baseline Characteristics**

| | Prevalent AFib N=135 | No AFib N=620 | p-value |
|---|---|---|---|
| **Demographics** | | | |
| Age, years, mean (SD) | 65.2 (7.0) | 64.0 (7.0) | 0.07 |
| Male Sex, n (%) | 63 (46.7%) | 335 (54.0%) | 0.12 |
| Race, n (%) | | | 0.92 |
| White | 113 (83.7%) | 525 (84.7%) | |
| Black | 5 (3.7%) | 25 (18.5%) | |
| Multi-Racial | 1 (0.7%) | 3 (0.5%) | |
| Unknown | 16 (11.9%) | 67 (10.8%) | |
| Ethnicity, n (%) | | | 0.35 |
| Non-Hispanic | 119 (88.1%) | 549 (88.5%) | |
| Hispanic | 0 | 4 (0.6%) | |
| Unknown | 16 (11.9%) | 67 (10.8%) | |
| Currently Smoke, n (%) | 34 (25.2%) | 215 (34.7%) | 0.03 |
| Pack Years, mean (SD) | 58.1 (33.7) | 54.1 (31.8) | 0.20 |
| **Cardiopulmonary Variables** | | | |
| $FEV_1$% predicted, mean (SD) | 0.67 (0.31) | 0.69 (0.30) | 0.54 |
| $FEV_1$% /FVC <70%, n (%) | 83 (61.5%) | 391 (63.1%) | 0.77 |
| DLco % predicted, mean (SD) | 0.57 (0.23) | 0.63 (0.25) | 0.02 |
| RV/TLC Ratio, mean (SD) | 0.42 (0.09) | 0.41 (0.09) | 0.56 |
| mMRC | 1.67 (1.36) | 1.41 (1.37) | 0.04 |
| % Emphysema | 0.61 (0.49) | 0.49 (0.50) | 0.008 |
| PA/PV Diameter Ratio | 0.94 (0.16) | 0.94 (0.16) | 0.85 |
| Heart Volume | 0.54 (0.18) | 0.51 (0.13) | 0.02 |
| Coronary Calcifications | 0.64 (0.91) | 0.65 (0.98) | 0.91 |
| Aorta Calcifications | 4.74 (5.64) | 3.95 (6.69) | 0.22 |
| **Comorbidities, n (%)** | | | |

| | | | |
|---|---|---|---|
| Myocardial Infarction | 6 (4.4%) | 24 (3.9%) | 0.58 |
| Diabetes | 15 (11.1%) | 31 (5%) | 0.002 |
| Angina | 22 (16.3%) | 53 (8.6%) | 0.007 |
| Heart Failure | 4 (3.0%) | 5 (0.8%) | 0.04 |
| Stroke | 6 (4.4%) | 27 (4.4%) | 0.96 |
| Thromboembolism | 14 (10.4%) | 26 (4.2%) | 0.004 |
| Anti-HTN Med | 79 (58.5%) | 228 (36.8%) | <0.001 |
| Hyperlipidemia | 79 (58.5%) | 302 (48.7%) | 0.02 |

*Abbreviations: Standard deviation (SD), forced expiratory volume in one sec ($FEV_1$), forced vital capacity (FVC), diffusion capacity of the lungs for carbon monoxide (DLco), Modified Medical Research Council (mMRC), residual volume/total lung capacity (RV/TLC), pulmonary artery/pulmonary vein (PA/PV) diameter, hypertension (HTN)*

#### *Association of Cardiopulmonary Variables with Prevalent AFib*

In univariate analysis, DLco, heart volume, percentage of emphysema, and mMRC all showed statistically significant associations with prevalent AFib (Table 2). These associations remained significant when adjusting for sex, age, number of pack years, BMI, heart failure, and anti-hypertensive medication use. $FEV_1$ and the presence of airflow obstruction were not significantly associated with prevalent AFib.

**Table 2. Association of Cardiopulmonary Variables with Prevalent AFib**

| | Univariate OR (95% CI) | Multivariable OR (95% CI) |
|---|---|---|
| $FEV_1$ % predicted | 0.83 (0.45-1.53) | 0.74 (0.39-1.43) |
| $FEV_1$% /FVC <70% | 0.94 (0.64-1.40) | 0.92 (0.61-1.37) |
| DLco % predicted<br><60%<br>≥60% | 0.40 (0.18-0.88)<br>1.60 (1.09-2.36)<br>Ref | 0.37 (0.16-0.83)<br>1.61 (1.09-2.38)<br>Ref |
| RV/TLC Ratio<br><40%<br>≥40% | 1.57 (0.40-6.17)<br>Ref<br>0.89 (0.60-1.31) | 1.40 (0.33-5.92)<br>Ref<br>0.79 (0.53-1.18) |
| mMRC | 1.15 (1.004-1.31) | 1.17 (1.02-1.35) |
| % Emphysema<br><20%<br>≥20% | 2.26 (0.87-5.83)<br>Ref<br>1.45 (1.27-1.66) | 2.77 (1.04-7.40)<br>Ref<br>1.80 (1.22-2.66) |
| PA/PV Diameter Ratio | 0.89 (0.26-2.99) | 0.79 (0.22-2.83) |
| Heart Volume | 4.67 (1.26-17.32) | 13.12 (2.32-74.17) |
| Right Coronary Calcifications | 2.30 (1.04-5.12) | 2.19 (0.94-5.13) |
| Left Coronary Calcifications | 0.93 (0.73-1.18) | 0.889 (0.68-1.16) |
| Coronary Calcifications | 0.99 (0.81-1.21) | 0.96 (0.77-1.20) |
| Aorta Calcifications | 1.02 (0.99-1.94) | 1.005 (0.98-1.03) |

*Abbreviations: odds ratio (OR), confidence interval (CI), forced expiratory volume in one sec ($FEV_1$), forced vital capacity (FVC), diffusion capacity of the lungs for carbon monoxide (DLco) , residual volume/total lung capacity (RV/TLC), Modified Medical Research Council (mMRC),*

***Cardiopulmonary Variables Improve Discrimination of AFib Risk Prediction Models***

In the prevalent AFib analysis, there was a statistically significant difference in mean $CHA_2DS_2$-VASc. For CHARGE-AF, there was no difference between the two groups. In the incident AFib analysis, there was no statistically significant difference for both risk scores (Table 3).

**Table 3. Mean AFib Risk Prediction Scores**

| | Prevalent AFib N=135 | No AFib N=620 | p-value | Incident AFib N=39 | No AFib N=581 | p-value |
|---|---|---|---|---|---|---|
| $CHA_2DS_2$-VASc | 2.28 | 1.68 | <0.001 | 1.56 | 1.69 | 0.55 |
| CHARGE-AF | 12.44 | 12.35 | 0.38 | 12.40 | 12.35 | 0.74 |

When conducting the discrimination analysis for prevalent AFib, the C-statistic was greater for $CHA_2DS_2$-VASc than CHARGE-AF, indicating it as a better risk predictor in our study population. This improved with the sequential addition of our exposure variables. Model 4 had the greatest improvement of discrimination, most notably for CHARGE-AF; this model incorporated DLco, heart volume, degree of emphysema, and mMRC score.

In the discrimination analysis for incident AFib, the CHARGE-AF C-statistic was greater than that of $CHA_2DS_2$-VASc, and the greatest improvement in risk discrimination was observed when cardiopulmonary variables were added to the $CHA_2DS_2$-VASc model (Table 4 and Figure 1).

**Table 4. Discrimination of AFib Risk Prediction Scores Updated with Cardiopulmonary Variables**

| | Prevalent AFib C-Statistic (95% CI) | p-value* | Incident AFib C-Statistic (95% CI) | p-value* |
|---|---|---|---|---|
| **$CHA_2DS_2$-VASc** | 0.62 | | 0.51 | |
| [a]Model 1 | 0.63 (0.57-0.68) | 0.43 | 0.65 (0.58-0.73) | 0.01 |
| [b]Model 2 | 0.65 (0.60-0.70) | 0.13 | 0.66 (0.58-0.74) | 0.01 |
| [c]Model 3 | 0.66 (0.60-0.71) | 0.11 | 0.68 (0.61-0.75) | 0.001 |
| [d]Model 4 | 0.66 (0.60-0.71) | 0.10 | 0.70 (0.63-0.77) | <0.001 |
| **CHARGE-AF** | 0.53 | | 0.54 | |
| [a]Model 1 | 0.59 (0.53-0.66) | 0.11 | 0.53 (0.42-0.64) | 0.76 |
| [b]Model 2 | 0.63 (0.56-0.69) | 0.04 | 0.54 (0.45-0.63) | 0.88 |
| [c]Model 3 | 0.62 (0.56-0.69) | 0.04 | 0.58 (0.49-0.68) | 0.35 |
| [d]Model 4 | 0.64 (0.57-0.70) | 0.02 | 0.62 (0.53-0.71) | 0.14 |

*Abbreviations: confidence interval (CI)*

**p-value reflects comparison between each Model to the corresponding AF risk prediction model, $CHA_2DS_2$-VASc or CHARGE-AF*

*Each model incorporates either $CHA_2DS_2$-VASc or CHARGE-AF plus the following:*

*[a]Model 1: DLco % predicted*

*[b]Model 2: DLco % predicted, heart volume*

*[c]Model 3: DLco % predicted, heart volume, % emphysema $\geq$ 20%,*

*[d]Model 4: DLco % predicted, heart volume, % emphysema $\geq$ 20%, mMRC score*

## Discussion

In our analysis, we found that lower DLco, larger heart volume, greater percentage of emphysema, and worse mMRC scores are associated with an increased risk of AFib in our tobacco-exposed cohort. We found that $CHA_2DS_2$-VASc was an overall better predictor of prevalent AFib than CHARGE-AF in our study population. The addition of cardiopulmonary physiologic and radiographic variables to these risk prediction models improves risk discrimination in all domains. Specifically, in prevalent AFib, the incorporation of the Model 4 variables (DLco, heart volume, percentage of emphysema, and mMRC score) improved risk discrimination of CHARGE-AF to a greater degree compared to $CHA_2DS_2$-VASc. In contrast, in incident AFib, the addition of these variables improved risk discrimination of $CHA_2DS_2$-VASc to a greater degree than CHARGE-AF. Both risk prediction models alone had overall worse discrimination than previously validated studies[7,8], likely due to differences in study populations, with our study population having a significantly greater percentage of smokers and hypertension compared to those populations studied in $CHA_2DS_2$-VASc and CHARGE-AF.

Large cohort studies, including the Copenhagen City Heart Study, the Atherosclerosis Risk in Communities (ARIC) cohort study, and the Malmo Preventative project show that a lower $FEV_1$ is associated with an increased risk of developing AFib[5]. While $FEV_1$ was not shown to be statistically significant in our study population, we found that previously unstudied variables, including DLco, percentage of emphysema, and mMRC are associated with prevalent AFib. Emphysema leads to DLco impairment and hypoxia which is known to contribute to AFib risk[4,5]. Atrial dilation likewise associates with AFib[5], and while not feasible to measure in our cohort, total heart volume was associated with risk of Afib in our cohort.

The addition of cardiopulmonary variables to $CHA_2DS_2$-VASc and CHARGE-AF greatly improved risk discrimination, most pronounced in $CHA_2DS_2$-VASc predicting incident AFib. While there are many AFib risk prediction models available, CHARGE-AF has been shown to have the best success in predicting AFib development in 5 years[19,20]. Both $CHA_2DS_2$-VASc and CHARGE-AF scores are comprised of demographic data and cardiac comorbidities. Neither includes lung-related variables and, although both were derived from a diverse patient population of > 10,000 individuals, they did not assess lung-related factors other than smoking status[7,8]. Therefore, whether these scores are generalizable to a COPD population is unclear, and their relative low performance in our tobacco-exposed cohort would suggest that they are not. We have shown that the incorporation of additional cardiopulmonary variables to these validated risk scores improves AFib risk prediction in a COPD population.

Our analysis has several limitations to note. AFib was assessed via self-report rather than through electronic health record (EHR) review or EKG documentation. Since AFib is often asymptomatic and paradoxical, our number of participants with AFib was likely an under-estimate of the true prevalence. In our logistic regression analyses, we only assessed the association of our cardiopulmonary variables with prevalent AFib due to a smaller sample size in the incident AFib group and concern for survivorship bias as we found that our incident AFib group had a higher $FEV_1$, less pack year history, and fewer obstructive PFTs. This was also affected by a high drop-out rate. While we did not validate our findings in a separate cohort, this likely did not affect study results as the $CHA_2DS_2$-VASc and CHARGE-AF have already been validated in prior cohorts. Finally, our CT variables were created by an AI research algorithm and therefore may not be generalizable to a clinical population.

## Conclusion

We identified cardiopulmonary factors associated with an increased risk of AFib in a tobacco-exposed cohort. The incorporation of lung function, CT parameters, and symptom scores in validated AFib prediction models may improve AFib risk discrimination in our chronic lung disease populations.

## Declarations

**Ethics approval and consent to participate:** This study was conducted in accordance with the Declaration of Helsinki and good clinical practice guidelines. The SCCOR study was approved by the University of Pittsburgh ethics and review board. All participants gave written informed consent.

**Consent for publication:** Not applicable

**Availability of data and materials:** Data can be made available upon request by emailing the corresponding author.

**Competing Interests:** The authors declare no competing interests.

**Funding:** This study was supported by the National Institute of Health (NIH) National Heart, Lung and Blood Institute (NHLBI) grants 1R01HL128289 (J.B.) and P50HL084948 (F.C.S.).

**Authors' contributions:** All authors significantly contributed to either the study conception or design, data collection, statistical analysis, and/or manuscript creation. All authors reviewed the final manuscript and provided approval.

**Acknowledgements:** Not applicable.

2023;10(5):051806. 10.1117/1.JMI.10.5.051806.

17. American College of Cardiology/American Heart Association Joint Committee on Clinical Practice Guidelines. 2023 ACC/AHA/ACCP/HRS guideline for the diagnosis and management of atrial fibrillation: A report of the American College of Cardiology/American Heart Association Joint Committee on Clinical Practice Guidelines. *J Am Coll Cardiol*. 2023;81(6):e247-e350. 10.1016/j.jacc.2022.10.017
18. StataCorp. Stata Statistical Software: Release 18. College Station. TX: StataCorp LLC; 2023.
19. Christophersen IE, Yin X, Larson MG, et al. A comparison of the CHARGE-AF and the $CHA_2DS_2$-VASc risk scores for prediction of atrial fibrillation in the Framingham Heart Study. Am Heart J. 2016;178:45–54. 10.1016/j.ahj.2016.05.004.
20. Hulme OL, Khurshid S, Weng L-C, et al. Development and validation of a prediction model for atrial fibrillation using electronic health records. JACC Clin Electrophysiol. 2019;5(11):1331–41.

## Figures

**Figure 1. Discrimination of An Updated Model for Incident AFib Risk Prediction**

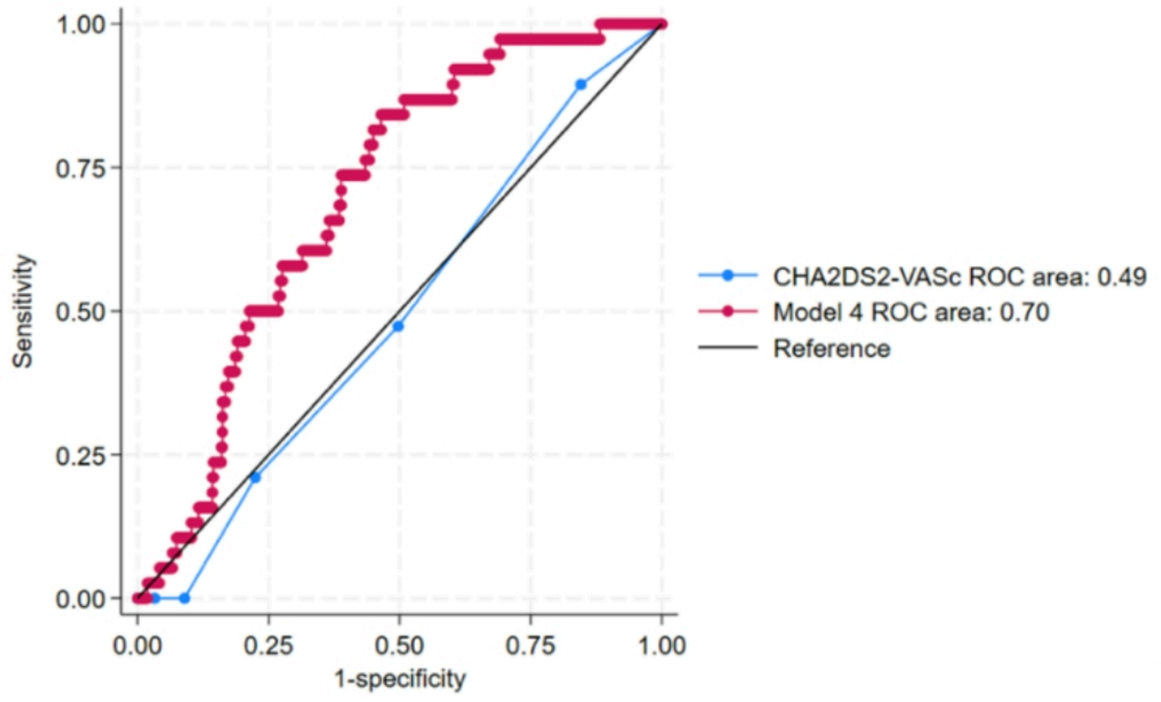

**Figure 1**

See image above for figure legend
